# Altered gene and protein expression in liver of the obese spontaneously hypertensive/NDmcr-cp rat

**DOI:** 10.1186/1743-7075-9-87

**Published:** 2012-09-21

**Authors:** Jie Chang, Shinji Oikawa, Gaku Ichihara, Yui Nanpei, Yasuhiro Hotta, Yoshiji Yamada, Saeko Tada-Oikawa, Hitoshi Iwahashi, Emiko Kitagawa, Ichiro Takeuchi, Masao Yuda, Sahoko Ichihara

**Affiliations:** 1 Graduate School of Regional Innovation Studies, Mie University, 1577 Kurimamachiya-cho, Tsu 514-8507, Japan; 2 Department of Environmental and Occupational Health, Nagoya University Graduate School of Medicine, Nagoya, Japan; 3 Department of Molecular and Environmental Medicine, Mie University Graduate School of Medicine, Tsu, Japan; 4 Department of Human Functional Genomics, Life Science Research Center, Mie University, Tsu, Japan; 5 Health Technology Research Center, National Institute of Advanced Industrial Science and Technology, Tsukuba, Japan; 6 Department of Engineering, Nagoya Institute of Technology, Nagoya, Japan; 7 Department of Medical Zoology, Mie University Graduate School of Medicine, Tsu, Japan; 8 Graduate School of Applied Biological Sciences, Gifu University, Gifu, Japan; 9 Applied Science, Roche Diagnostics, Tokyo, Japan

**Keywords:** Metabolic syndrome, Pathophysiology, Microarray analysis, Proteomics analysis, Obesity, Liver

## Abstract

**Background:**

It is difficult to study the mechanisms of the metabolic syndrome in humans due to the heterogeneous genetic background and lifestyle. The present study investigated changes in the gene and protein profiles in an animal model of the metabolic syndrome to identify the molecular targets associated with the pathogenesis and progression of obesity related to the metabolic syndrome.

**Methods:**

We extracted mRNAs and proteins from the liver tissues of 6- and 25-week-old spontaneously hypertensive/NIH –corpulent rat SHR/NDmcr-cp (CP), SHR/Lean (Lean) and Wistar Kyoto rats (WKY) and performed microarray analysis and two-dimensional difference in gel electrophoresis (2D-DIGE) linked to a matrix-assisted laser desorption ionization time-of-flight tandem mass spectrometry (MALDI-TOF/TOF MS).

**Results:**

The microarray analysis identified 25 significantly up-regulated genes (*P* < 0.01; log_10_ > 1) and 31 significantly down-regulated genes (*P* < 0.01; log_10_ < −1) in 6- and 25-week-old CP compared with WKY and Lean. Several of these genes are known to be involved in important biological processes such as electron transporter activity, electron transport, lipid metabolism, ion transport, transferase, and ion channel activity. MALDI-TOF/TOF MS identified 31 proteins with ±1.2 fold change (*P* < 0.05) in 6- and 25-week-old CP, compared with age-matched WKY and Lean. The up-regulated proteins are involved in metabolic processes, biological regulation, catalytic activity, and binding, while the down-regulated proteins are involved in endoplasmic reticulum stress-related unfolded protein response.

**Conclusion:**

Genes with significant changes in their expression in transcriptomic analysis matched very few of the proteins identified in proteomics analysis. However, annotated functional classifications might provide an important reference resource to understand the pathogenesis of obesity associated with the metabolic syndrome.

## Background

The World Health Organization (WHO) has reported that once considered a problem only in high income countries, overweight and obesity are now dramatically on the rise in low- and middle-income countries, particularly in urban settings [1]. Overweight and obesity related to the metabolic syndrome are also major risk factors for a number of chronic diseases, including diabetes, cardiovascular diseases, and cancer [2,3]. The metabolic syndrome is defined as a cluster of abdominal obesity, atherogenic dyslipidemia (hypertriglyceridemia, hypo-HDL cholesterolemia, both foster plaque buildup in arterial walls), hypertension, and hyperglycemia (the body cannot properly use insulin or blood sugar) [4]. The etiology of the metabolic syndrome is complex, and is determined by the interplay of both genetic and environmental factors [5]. Several environmental factors, including smoking, high-calorie diet, and physical inactivity influence the development of the metabolic syndrome [6].

The spontaneously hypertensive/NIH –corpulent rat (SHR/NDmcr-cp) is a new genetically obese strain that spontaneously develops hypertension, hyperlipidemia, and non-insulin-independent diabetes mellitus [7]. The SHR/NDmcr-cp is a sub-strain of the SHR/N-cp. It has a genetic background from the spontaneously hypertensive rat (SHR) and carries nonsense mutation of leptin receptor derived from obese Koletsky rat [8]. Since the phenotype of the SHR/NDmcr-cp is similar to that of patients with the metabolic syndrome, the SHR/NDmcr-cp is thought to be one of the most suitable animal models of the metabolic syndrome [9]. Given that studying SHR/NDmcr-cp would help to clarify some of the pathophysiological mechanisms of this syndrome, we used this rat model of the metabolic syndrome in the present study.

Using microarray and proteomics analyses, the present study was designed to identify genes and proteins in the liver tissues with altered expression in the SHR/NDmcr-cp rats and to find possible molecular targets associated with the pathogenesis or progression of obesity related to the metabolic syndrome.

## Methods

### Animals

SHR/NDmcr-cp (cp/cp) (CP), SHR/Lean (Lean), and Wistar Kyoto rats (WKY) were purchased from the Disease Model Cooperative Research Association (Kyoto, Japan) and used for this study at 6- and 25-weeks of age. All animals were fed normal diet and housed in a temperature-controlled environment (25°C) with a 12-hour light–dark cycle. The investigation conformed to the Guide for the Care and Use of Laboratory Animals published by the US National Institutes of Health (NIH Publication No. 85–23, revised 1996) and was approved by the Committee on Laboratory Animals Utilization of Mie University.

### Measurement of blood pressure and biochemical tests

Systolic blood pressure (SBP) was measured in six conscious rats per group by the tail-cuff method as described previously [10]. Blood was collected from anesthetized rats, transferred to a chilled tube containing heparin, and centrifuged. Plasma was stored at −80°C until analysis. Serum levels of triglyceride, total-cholesterol, and glucose were measured at SRL (Tokyo, Japan) (*n* = 6 for 6- and 25-week-old rats).

### Preparation of RNA and microarray analysis

The liver tissues were obtained from 6- and 25-week-old rats (n = 6, each), immediately frozen in liquid nitrogen, and stored at −80°C. Total RNA was extracted from about 250 mg of liver tissue using the RNeasy Mini Kit (Qiagen, Valencia, CA) according to the instructions provided by the manufacturer. Total RNA concentration was quantified by spectrophotometry (ND-1000, NanoDrop Technologies, Wilmington, DE). Then, a search for genome-wide expression changes was conducted using oligo nucleotide microarray (Whole Rat Genome microarray 22 K, Agilent Technologies, Santa Clara, CA) in *1)* WKY and CP at 6 weeks of age, *2)* Lean and CP at 6 weeks of age, *3)* WKY and CP at 25 weeks of age, and *4)* Lean and CP at 25 weeks of age. Two microarray slides were used for each comparison in two different RNA samples from each strain.

### Hybridization and microarray scanning

Reverse transcription labeling and hybridization were conducted using the Agilent 60-mer Oligo microarray (Agilent Technologies), according to the instructions provided by the manufacturer. Briefly, total RNA of 500 ng prepared from the individual rat liver tissue samples was used for cDNA synthesis using a T7 promoter primer with Agilent low RNA input fluorescent linear amplification kit. The cRNA was synthesized using T7 RNA polymerase. The reaction was carried out in a solution containing 50 mM dATP/dGTP/dTTP, 25 mM dCTP, 25 mM cyanine 3 (Cy3)-dCTP (for WKY or Lean sample) or cyanine 5 (Cy5)-dCTP (for CP sample) (NEL580 and 581, Perkin Elmer Life Science, Waltham, MA) and 400 U MMLV reverse transcriptase at 42°C for 1 hr. The labeled cRNA samples were purified using Qiagen’s RNA mini spin columns. Every cyanine-labeled cRNA (0.75 μg) was individually hybridized to a rat Agilent Oligo 22 k microarray slide. Hybridization was carried out in 22 mL of a hybridization mixture containing cDNA probes at 65°C for 17 hr. The glass slides were then washed with 0:5 × SSC and 0.01% sodium dodecyl sulfate (SDS) at room temperature for 5 min, and with 0:06 × SSC at room temperature for 2 min. Immediately after removing the wash buffer by centrifugation, the glass slides were scanned with an Agilent DNA MicroArray Scanner (Agilent Technologies) containing a 532 nm laser for Cy3 measurement and a 635 nm laser for Cy5 measurement.

### Quantitative RT-PCR analysis

To confirm the results of microarray analysis, total RNA extracted from the liver tissue was subjected to quantitative reverse transcription and polymerase chain reaction (RT-PCR) analysis with primers specific for mRNAs encoding ELOVL family member 6, elongation of long chain fatty acids (*Elovl6*) and fatty acid synthase (*Fasn*) (*n* = 6 for 6- and 25-week-old rats). Amplification was carried out in a reaction volume of 25 μl, containing: 12.5 μl of FastStart Universal Probe Master (2 ×) (Roche Applied Science, Mannheim, Germany), 200 nM of each primer, 100 nM of Universal ProbeLibrary probe (Roche Applied Science) and 2 μl of DNA template. Thermal cycling was initiated with 2 min incubation at 50°C, followed by 10 min denaturation at 95°C. Then 40 cycles of 95°C for 15 s and 65°C for 60 s were applied. The mRNA for β-actin was used as an internal control.

### Preparation of protein samples

Frozen liver tissues were also homogenized in lysis buffer (30 mM Tris–HCl, 7 M urea 2 M thiourea, 4% w/v CHAPS, and a protease inhibitor cocktail, pH 8.5). After incubation for 60 min on ice, homogenates were centrifuged at 30,000 × *g* for 30 min at 4°C and the supernatant was collected. Protein concentration was determined in the supernatant by the Bradford assay (Bio-rad Laboratories, Hercules, CA), using bovine serum albumin as a standard [11].

### Two-dimensional fluorescence difference gel electrophoresis (2D-DIGE)

For 2D-DIGE, 25 μg of each sample were labeled with 200 pmol of amine-reactive cyanine dyes, Cy3 or Cy5 developed for fluorescence 2D-DIGE technology (GE Healthcare, Buckinghamshire, UK) (*n* = 4 for 6- and 25-week-old rats) [12]. Internal pools were generated by combining equal amounts of all samples and were labeled with Cy2. Then, two-dimensional gel electrophoresis (2DE) was performed, as described previously [13]. After 2DE, cyanine-labeled proteins were visualized directly by scanning, using the Typhoon 9400 imager (GE Healthcare) in fluorescence mode. The differential in-gel analysis module of the DeCyder software (GE Healthcare) was used for automatic detection followed by editing of protein spots. The same software was used for abundance measurements for each gel by comparing normalized volume ratios of individual spots from Cy3- or Cy5-labeled samples to corresponding Cy2-signals from the pooled samples (internal standard) [14]. Thereafter, all gel comparisons and initial screening type statistical analyses were performed with the biological variation analysis module.

### Protein identification

After image analysis, gels containing the additional load of unlabeled proteins from the liver tissues were stained with Colloidal Coomassie Brilliant Blue G (GE Healthcare) and matched to the fluorescent 2D-DIGE images. Selected spots were picked and in-gel digestion of protein samples was performed using the protocol described in detail previously [13,15]. Mass analysis of peptide mixtures was performed using a matrix-assisted laser desorption ionization time-of-flight tandem mass spectrometry (MALDI-TOF/TOF MS; 4800 *Plus* MALDI TOF/TOF^TM^ Analyzer, Applied Biosystems, Foster City, CA) operating in positive-ion reflector mode. The Paragon Method was applied for protein database search, using Protein Pilot software (Applied Biosystems) to identify excised proteins.

### Western blot analysis

To confirm the results of proteomic analysis, western blot analysis was conducted. Samples (*n* = 4 in each group) containing 10 μg of liver tissue proteins were separated by 12% SDS-PAGE and electroblotted onto polyvinylidene difluoride (PVDF) membranes. The membranes were incubated with rabbit polyclonal antibodies to mouse 10-formyltetrahydrofolate dehydrogenase (FTHFD) (Abcam, Cambridge, UK) at a 1:10,000 dilution and to human carbonic anhydrase III (CA3) (Abcam) at a 1:1,000 dilution. The formed immunocomplexes were visualized by enhanced chemiluminescence (GE Healthcare) using ChemiDox XRS-J (Bio-rad Laboratories). Mouse anti-β-actin monoclonal antibody (Sigma, St Louis, MO) at a 1:10,000 dilution was used as a loading control. The density of the bands was quantified by Quantity One v3.0 software (Bio-rad Laboratories). Protein expression levels were normalized relative to the level of β-actin protein in the same tissue sample.

### Statistical analysis

Microarray data analysis was carried out using GeneSpring ver. 7.3.1 software (Agilent Technology) [16,17]. The detected signals were normalized using GeneSpring normalization algorithms. In each comparison, genes were filtered based on minimum ±1.0 of log ratio (WKY vs. CP or Lean vs. CP) and P < 0.01 using Student’s test. Gene ontology (GO) analysis was performed using GeneSpring (Agilent Technology). With regard to the data of proteomics analysis, the differential in-gel analysis module of the DeCyder software (GE Healthcare) was used for automatic detection of protein spots and for abundance measurements of each gel by comparing the normalized volume ratios of individual spots [14]. Two-tailed Student’s *t*-test was performed to determine the differences between paired groups (WKY vs. CP or Lean vs. CP) using the biological variation analysis module of DeCyder software (GE Healthcare). Data are presented as mean ± SEM. Differences among physiological and biochemical parameters and the quantitated data of both RT-PCR and western blot analyses were evaluated for statistical significance by one-way analysis of variance followed by Dunnett’s post hoc test. Statistical analyses were performed using the JMP 8.0 software (SAS Institute Inc., Cary, NC). A *P* value of < 0.05 was considered statistically significant.

## Results

### Changes in body weight, systolic blood pressure, and biochemical parameters

Body weight and liver weight were significantly greater in CP than in WKY and Lean at 6 and 25 weeks of age (Table 1). CP and Lean became hypertensive from 6 weeks of age, and there was no significant difference in systolic blood pressure at the two ages between CP and Lean (Table 1). At 6 weeks of age, there was no significant difference in triglyceride and glucose levels among the three groups, however, at 25 weeks of age, these levels were significantly higher in CP than in both WKY and Lean (Table 1). These results indicate the development of early stages of the metabolic syndrome at 6 weeks of age and the chronic stage of the syndrome at 25 weeks of age in CP. There were no significant differences in triglyceride and glucose levels between WKY and Lean at the two ages (Table 1). Therefore, we used WKY and Lean as the controls to identify the possible molecular targets associated with the pathogenesis or progression of obesity associated with the metabolic syndrome.

**Table 1:** Body and liver tissue weights, blood pressure, and biochemical data in 6- and 25-week-old rats

	**6 weeks of age**			**25 weeks of age**		
	**WKY**	**Lean**	**CP**	**WKY**	**Lean**	**CP**
Body weight (g)	140.2 ± 1.1	147.5 ± 1.2	228.5 ± 1.3*†	422.8 ± 5.2	413.9 ± 4.1	578.4 ± 6.8*†
Liver weight (g)	7.2 ± 0.9	7.5 ± 0.6	12.5 ± 1.1*†	12.8 ± 1.2	13.9 ± 1.1	28.4 ± 2.8*†
Blood pressure (mmHg)	101.5 ± 1.2	179.5 ± 1.4*	173.3 ± 1.2*	108.7 ± 1.8	208.7 ± 2.1*	191.2 ± 4.0*
Triglyceride (mg/dl)	65.8 ± 7.7	65.2 ± 3.7	93.3 ± 13.1	62.8 ± 12.5	61.8 ± 5.5	816.3 ± 51.6*†
Non-fasting glucose (mg/dl)	178.4 ± 7.9	173.7 ± 7.6	189.2 ± 4.0	160.7 ± 5.9	186.2 ± 34.5	278.2 ± 26.7*†

Data are mean ± SEM of six rats per group.

*P < 0.05, compared with the corresponding value for WKY;

† *P < 0.05*, compared with the corresponding value for Lean.

WKY; Wistar Kyoto rats, Lean; spontaneously hypertensive rats (SHR/lean), CP; SHR/NDmcr-cp (cp/cp).

### Gene expression profiles by microarray analysis

After the experiments of microarray analysis, all hybridization spots on the image were quantified. The data of the fluorescence intensity were converted into log_10_ values. Genes with significantly different expression levels (P < 0.01), relative to WKY and Lean, were extracted for further analysis. On the oligoDNA microarray, 253 and 125 genes were significantly up-regulated (*P* < 0.01 and log_10_ > 1) in 6-week-old CP compared with age-matched WKY and Lean. Furthermore, 244 and 97 genes were down-regulated (*P* < 0.01 and log_10_ < −1) in 6-week-old CP compared with age-matched WKY and Lean. We also found 163 and 149 significantly up-regulated genes and 184 and 162 down-regulated genes in 25-week-old CP compared with 25-week-old WKY and Lean. Among these, 25 genes were significantly up-regulated (Additional file 1: Table S1) and 31 genes were significantly down-regulated (Additional file 1: Table S2) in the liver tissues of 6- and 25-week-old CP, compared with age-matched WKY and Lean. The results of microarray analysis were confirmed by quantitative RT-PCR analysis. The mRNA levels of Elovl6 and Fasn were greater in of 6- and 25-week-old CP, compared with age-matched control (WKY and Lean) (Additional file 2: Figure S1).

### Functional categories of genes with altered expression in CP

To understand their biological roles, the genes with significant changes in expression detected in CP by microarray analysis were assigned to established GO classification by GeneSpring ver. 7.3.1. In GO classification, we found three aberrant GO terms (*p <* 0.01) in up-regulated genes, electron transporter activity, electron transport, and lipid metabolism and five GO terms (*p <* 0.01) in down-regulated genes electron transporter activity, electron transport, ion transport, transferase, and ion channel activity (Table 2). For the results of Kyoto Encyclopedia of Genes and Genomes (KEGG) molecular pathway analysis, genes with significant change in expression in CP by microarray analysis were also assigned to KEGG molecular pathway by GeneSpring. In KEGG molecular pathway analysis, 10 or 6 predicted pathways were found in the up-regulated and down-regulated genes, respectively (Table 2). The genes with significant change in expression in CP by microarray analysis were also imported into the PANTHER database. The PANTHER classification system indicated that the up-regulated and down-regulated genes in CP can be classified into eight groups according to their functional properties: *1)* lipid metabolism, *2)* carbohydrate metabolism, *3)* protein metabolism, *4)* cellular amino acid metabolism, *5)* immune system, *6)* cell adhesion, *7)* signal transduction, and *8)* others (Additional file 1: Table S1 and Additional file 1: Table S2).

**Table 2:** List of entities identified by GO and the pathways identified by KEGG of significantly up-regulated and down-regulated genes in liver tissues in 6- and 25-week-old CP

**GO accession**	**GO Term**	***P*value**
*Up-regulation*		
GO:0005489	electron transporter activity	7.31E-06
GO:0006118	electron transport	7.42E-06
GO:0006629	lipid metabolism	5.07E-05
*Down-regulation*		
GO:0005489	electron transporter activity	1.00E-03
GO:0006118	electron transport	1.01E-03
GO:0006811	ion transport	1.55E-03
GO:0010740	transferase	3.29E-03
GO:0005216	ion channel activity	4.86E-03
**Predicted pathway**		***P* value**
*Up-regulation*		
Carbon fixation		2.54E-06
Pentose phosphate pathway		1.86E-04
Pyruvate metabolism		4.07E-04
Glutathione metabolism		4.07E-04
Fructose and mannose metabolism		5.27E-04
Metabolism of xenobiotics by cytochrome P450		7.61E-04
Fatty acid metabolism		9.78E-04
Glycolysis-Gluconeogenesis		1.04E-03
Arachidonic acid metabolism		1.49E-03
Fatty acid biosynthesis		5.44E-03
*Down-regulation*		
gamma-hexachlorocyclohexane degradation		6.48E-07
Metabolism of xenobiotics by cytochrome P450		7.14E-06
Linoleic acid metabolism		2.36E-06
PPARγ signaling pathway		2.78E-03
Methionine metabolism		7.57E-03
Glyoxylate and dicarboxylate metabolism		7.57E-03

PPARγ: peroxisome proliferator-activated receptor γ.

### Identification of proteins with significant change in expression levels

To investigate the changes in protein expression levels in the liver, proteins extracted from the liver tissues of 6- and 25-week-old WKY, Lean, and CP were subjected to comparative analysis by 2D-DIGE. From approximately 2000 (2295) spots detected in each gel, 307 protein spots were found to be modified in 6-week-old CP compared with age-matched WKY and Lean, whereas 407 spots were found to be modified in 25-week-old rats. Among these, 63 spots were significantly modified in 6- and 25-week-old CP according to the DeCyder software analysis with an absolute ratio of more than 1.2-folds (p < 0.05), compared with age-matched WKY and Lean.

**Figure 1:**
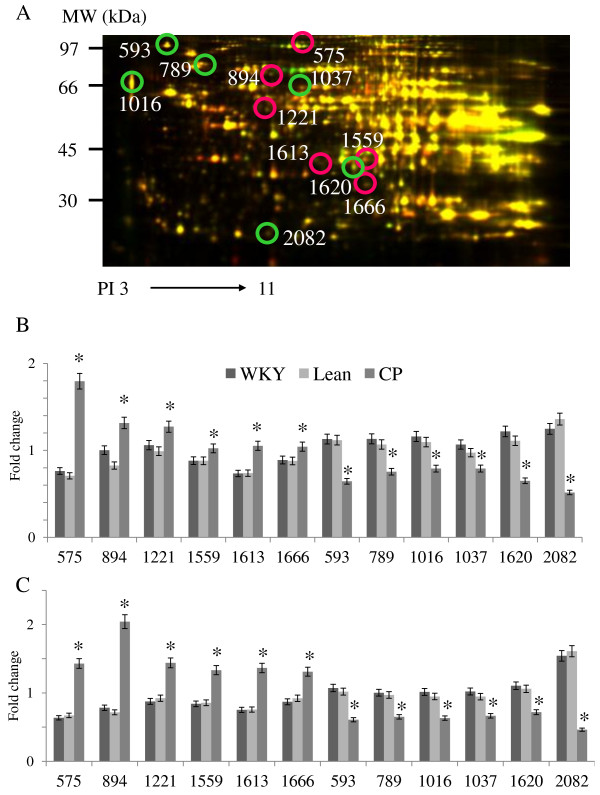
**A representative 2D-DIGE image of liver lysates from 25-week-old Lean and CP.** (**A**) The proteins (40 μg each) were labeled with Cy3 and Cy5 dyes, mixed and subjected to 2D-DIGE analysis. We selected significantly different spots of 6- and 25-week-old CP, relative to age-matched control (WKY and Lean) rats. The representative 12 quantified expression levels of each strain at 6 (**B**) and 25 weeks of age (**C**) were expressed as mean ± SEM from four rats per group. *P < 0.05, compared with the corresponding value for WKY or Lean.

These differential spots were cut from the gels, digested and applied to MALDI-TOF-MS analysis. The latter identified 31 spots (Figure 1), which included 13 spots with significantly up-regulated expression in 6- and 25-week-old CP, compared with age-matched WKY and Lean (Table 3), and 18 spots significantly down-regulated in 6- and 25-week-old CP, compared with age-matched WKY and Lean (Table 3). The peptide mass peaks were compared with those in the protein database using Paragon Method. Western blot analysis was also performed to confirm the results of proteomics analysis and the findings (Additional file 2: Figure S2A) were consistent with those of proteomic results; the expression of FTHFD was significantly up-regulated while that of CA3 was significantly down-regulated in 6- and 25-week-old CP compared with age-matched control (WKY and Lean) (Additional file 2: Figure S2B).

**Table 3:** List of liver tissue proteins with significant spot changes identified in 6- and 25-week-old CP

**Spot no.**	**Protein name**	**% Cover**	**Peptides (95%)**	**Fold change**			
				**6-week-old**		**25-week-old**	
				**WKY-CP**	**Lean-CP**	**WKY-CP**	**Lean-CP**
*Up-regulated proteins*							
458	Pyruvate carboxylase	3.1	3	1.15	1.15	1.27	1.22
561	Staphylococcal nuclease domain-containing protein 1	6.4	5	1.41	1.43	2.19	2.35
575	10-formyltetrahydrofolate dehydrogenase	12.4	7	2.35	2.54	2.25	2.13
579	10-formyltetrahydrofolate dehydrogenase	10.3	5	1.91	1.89	1.96	1.84
595	Dimethylglycine dehydrogenase	8.3	4	1.22	1.27	1.55	1.77
894	Dihydrolipoyllysine-residue acetyltransferase component of pyruvate dehydrogenase complex	9.7	3	1.46	1.59	2.61	2.85
1221	Glutathione synthetase	19.2	5	1.2	1.28	1.64	1.56
1559	Glycerol-3-phosphate dehydrogenase [NAD+]	21	6	1.16	1.16	1.77	1.54
1568	N(G),N(G)-dimethylarginine dimethylaminohydrolase 1	25.6	6	1.35	1.37	2.31	1.91
1604	Pyruvate dehydrogenase E1 component subunit beta	25.4	7	1.18	1.25	1.6	1.68
1613	Malate dehydrogenase	15	5	1.43	1.43	1.81	1.8
1666	Ketohexokinase	30.2	6	1.2	1.19	1.51	1.42
1925	Glutathione S-transferase kappa 1	9.3	2	1.57	1.6	1.93	1.6
*Down-regulated proteins*							
273	Hypoxia up-regulated protein 1	17	5	−1.53	−1.42	−1.55	−1.35
593	Endoplasmin	12.7	8	−1.75	−1.73	−1.76	−1.68
789	Protein disulfide-isomerase A4	8.6	5	−1.5	−1.42	−1.54	−1.49
819	78 kDa glucose-regulated protein	14.3	6	−1.39	−1.27	−1.69	−1.47
820	78 kDa glucose-regulated protein	18.5	8	−1.44	−1.27	−1.82	−1.61
955	Alpha-2-HS-glycoprotein	15.3	3	−1.27	−1.2	−2.75	−3.15
1016	Calreticulin	33.2	9	−1.47	−1.39	−1.6	−1.5
1037	Protein disulfide-isomerase A3	19.4	6	−1.31	−1.23	−1.53	−1.42
1053	Protein disulfide-isomerase A3	14.7	5	−1.41	−1.28	−1.52	−1.48
1217	Keratin, type I cytoskeletal 18	11.1	4	−1.52	−1.44	−1.61	−1.63
1499	Farnesyl pyrophosphate synthase	9.9	3	−1.24	−1.22	−1.83	−1.76
1598	Regucalcin	24.4	7	−1.27	−1.19	−1.7	−1.85
1620	Sulfotransferase 1C1	30.3	6	−1.87	−1.71	−1.54	−1.48
1840	C-reactive protein	8.7	6	−1.23	−1.23	−1.89	−1.7
1860	Carbonic anhydrase 3	51.5	10	−4.27	−4.84	−2.12	−2.36
1862	Carbonic anhydrase 3	43.5	8	−2.69	−3.24	−1.72	−1.97
2082	Major urinary protein	37.6	6	−2.42	−2.64	−3.34	−3.48
2164	Cytochrome b5	55.2	7	−1.18	−1.18	−1.68	−1.52

Abbreviations as in Table 1.

### Functional categories of protein with altered expression in CP

**Figure 2:**
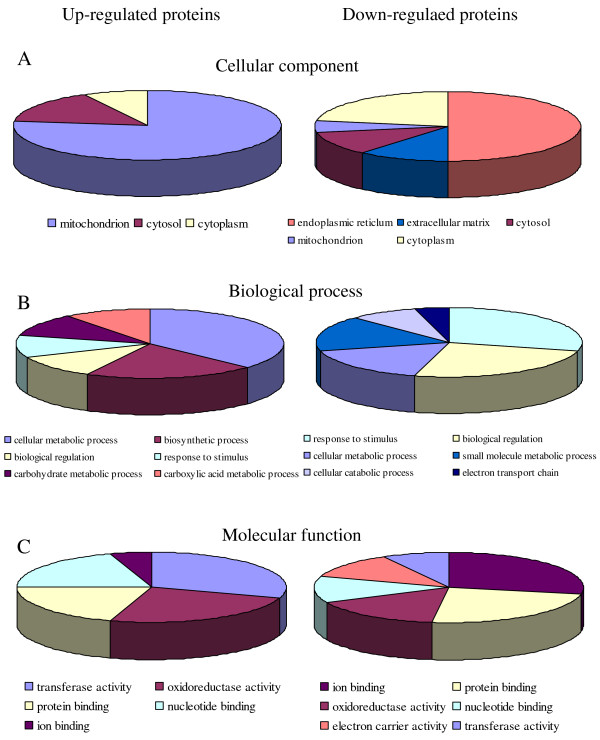
**Gene Ontology (GO) annotation of identified proteins.** The graphs show the percentages of corresponding GO terms to the total number of annotated proteins. The identified up-regulated or down-regulated proteins were mapped to GO at the three levels: cellular component (**A**), biological process (**B**), and molecular function (**C**).

To functionally annotate the identified proteins, we mapped them to GO at three levels: cellular component, biological process, and molecular function (Figure 2). The cellular component GO annotation revealed that most of the up-regulated GO annotated proteins were located at the mitochondrion while half of the down-regulated proteins were located at the endoplasmic reticulum (ER) (Figure 2A). The GO annotation for biological process showed that the up-regulated proteins were mainly involved in metabolic processes (Figure 2B). Furthermore, mapping for the molecular function showed that half of the up-regulated proteins belonged to catalytic activity and more than half of the down-regulated proteins belonged to binding (Figure 2C).

To understand their biological roles, proteins with significant changes in their expression by proteomics analysis were also imported into the PANTHER database. The PANTHER classification system indicated that the up-regulated and down-regulated genes in CP can be classified into six groups according to their functional properties: *1)* lipid metabolism, *2)* carbohydrate metabolism, *3)* protein metabolism, *4)* Nucleic acid metabolism, *5)* immune system process, and *6)* others (Additional file 1: Table S3). Additional file 1: Table S3 also shows the log ratio change of gene expression determined by microarray analysis for proteins identified by proteomics analysis in 6- and 25-week-old CP and age-matched control (WKY and Lean) rats.

## Discussion

Microarray analysis is a powerful tool for the evaluation of expression of many genes in various experimental systems and is particularly suited for the identification of target genes for transcription factors [18]. In the present study, we investigated simultaneous changes in gene and protein profiles using the metabolic syndrome rat model to identify the potential molecular targets associated with obesity- metabolic syndrome. The results of microarray and 2D-DIGE linked to MALDI-TOF-TOF analyses showed differential mRNA and protein expression in the liver tissue in the early and chronic stages of the metabolic syndrome.

In both microarray and proteomics analyses, the only significant change in 6- and 25-week-old CP, compared with the age-matched controls, was in sulfotransferase family 1C, membrane 1 (SULT1C1). SULT1C1 is expressed in the liver, lung, and intestine [19]. One subfamily of this enzyme, estrogen SULT, is expressed in subcutaneous adipose tissue and plays potential roles in glucose homeostasis and inflammation [20,21]. The members of this enzyme are reported to play important roles in not only the metabolism of drugs and xenobiotics but also the biotransformation of a variety of endogenous compounds [22]. Although the enzymatic properties and functional relevance of SULT1C1 remain unknown, SULT1C1 gene expression is reported to be down-regulated in isolated primary hepatocytes from livers of obese insulin-resistant Zucker rats [23], consistent with our results. In the present study, only a few of the genes with significant changes in microarray analysis were found to show matching change in the expression of their proteins in proteomics analysis (Tables 3, Additional file 1: Table S1 and Additional file 1: Table S2). Previous studies using high-throughput technologies (transcriptomic and proteomics analyses) demonstrated a positive correlation between transcript and protein levels for the majority of molecules [24,25]. Other studies, however, reported limited correlation between transcription and translation in mammals [26]. In the present microarray analysis, we only used genes with significant up-regulation (*P* < 0.01; log_10_ >1) or down-regulation (*P* < 0.01; log_10_ < −1) in 6- and 25-week-old CP, compared with WKY and Lean. Among the 27 identified proteins in proteomics analysis, the gene expression of 15 proteins was up-regulated or down-regulated by more than 1.5 fold in 6- and 25-week-old CP, compared with WKY and Lean (Additional file 1: Table S3). The different expression patterns noted in the two methodological approaches might arise from limitation of detection sensitivity or electrophoretic separation. Moreover, the discrepancy between the microarray and proteomics analyses might be due to differential regulation of translation, turnover, or alternative splicing.

The cellular component GO annotation revealed that the up-regulated GO annotated proteins were located in the mitochondria (90%) while the down-regulated proteins were mapped to the ER (65%). Our study showed down-regulation of both CRT and PDIA3/4. CRT is a highly versatile lectin-like chaperone involved in many cellular functions both inside and outside ER lumen [27]. The CRT-associated functions include acting as chaperone of nascent glycoproteins, regulator of Ca^2+^ homeostasis, cell adhesion, and inhibition of angiogenesis and tumor growth [28]. A highly adipogenic signal was detected in embryonic stem cells from *Crt*-deficient mice [29] and high glucose uptake and glycogen deposition were described in ventricular cardiomyocytes of these mice [30]. *Crt* deficiency also correlated with significant increases in insulin receptor expression, stability of glucose transporter 1 (GLUT1) expression, and in insulin-stimulated Akt phosphorylation and kinase activity, suggesting that the lack of CRT is associated with changes in insulin signaling and glucose metabolism [31-33]. While the potential effects of PDIA3 and PDIA4 are unknown, oxidative folding of glycoproteins in the ER and/or interaction with calnexin (CNX) and CRT could be affected [34]. PDI acts as a chaperone to promote oxidative refolding of non-monoglycosylated and reduces denatured lysozymes in the absence of CNX/CRT in vitro [35]. Furthermore, PDI is reported to interact with a specific set of glycoproteins that are recruited via its interactions with the lectins CNX/CRT [36,37]. ER stress has recently been implicated in the pathophysiology of obesity-related insulin resistance; however, what causes ER stress in obesity remains uncertain [38]. In our study, significant down-regulation of CRT and PDIA3/4 was noted in CP compared with WKY and Lean. Since the proteins involved in ER stress-related unfolded protein response were down-regulated, they may induce resistance to insulin and play an important role in the development of obesity associated with the metabolic syndrome.

## Conclusion

In the present study, we applied comparative genomic and proteomic analyses to identify the molecular targets in liver tissue associated with obesity of the metabolic syndrome. Microarray analysis identified 25 significantly up-regulated genes and 31 significantly down-regulated genes in the liver tissues of 6- and 25-week-old CP, compared with age-matched WKY and Lean. To gain further insight into the roles of these genes in biological processes, the genes with significantly change in their expression were classified according to their function. A large proportion of the genes were involved in electron transporter activity, electron transport, lipid metabolism, ion transport, transferase, and ion channel activity. Using proteomics approach, the up-regulated proteins were involved in metabolic process, biological regulation, catalytic activity, and binding. On the other hand, the down-regulated proteins were involved in ER stress-related unfolded protein response. These findings suggest that both groups of proteins might contribute to the development of obesity associated with the metabolic syndrome. These molecules may provide an important reference resource and be applied to the development of therapeutic targets for obesity associated with the metabolic syndrome.

## Abbreviations

2D-DIGE: Two-dimensional fluorescence difference gel electrophoresis; CA3: Carbonic anhydrase III; CNX: Calnexin; CRT: Calreticulin; ELOVL6: ELOVL family member 6, elongation of long chain fatty acids; ER: Endoplasmic reticulum; FASN: Fatty acid synthase; FTHFD: 10-formyltetrahydrofolate dehydrogenase; MALDI-TOF/TOF MS: Matrix-assisted laser desorption ionization time-of-flight tandem mass spectrometry; PDIA A3/4: Protein disulfide-isomerase A3/4; RT-PCR: Quantitative reverse transcription and polymerase chain reaction; SBP: Systolic blood pressure; SHR/NDmcr-cp: Spontaneously hypertensive/NIH –corpulent rat; WKY: Wistar Kyoto rats.

## Competing interests

The authors declare no conflict of interest.

## Authors' contribution

JC performed the experiments, analysis, and manuscript writing, SO and GI contributed to study design and manuscript writing, YN performed the proteomics analysis, YH performed the microarray analysis, YY and ST-O participated in data interpretation of proteomics analysis, HI, EK, IT, and MY participated in data interpretation of microarray analysis, SI participated in organization of the study, data interpretation, and preparation of the manuscript. All authors read and approved the final manuscript.

## Supplementary Material

Additional file 1: Table S1 List of significantly up-regulated genes in CP rats, compared with age-matched control rats (WKY and Lean). **Table S2.** List of significantly down-regulated genes in CP rats, compared with the age-matched control rats (WKY and Lean). **Table S3.** Comparison of gene expression in microarray analysis of proteins identified in proteomics analysis. Click here for file

Additional file 2: Figure S1 Expression of *Elovl6* and *Fasn* in liver tissues of 6- and 25-week-old WKY, Lean, and CP. Data are expressed relative to the mRNA expression of β-actin. All quantitative data are mean ± SEM values of six rats per group. *P < 0.05, compared with 6-week-old WKY and Lean; †*P* < 0.05, compared with 25-week-old WKY and Lean. **Figure S2.** Confirmation of the 2D-DIGE results by western blot analysis. (A) Representative immunoblot analysis of FTHFD, CA3, and β-actin (loading control) in liver tissues of representative 6- and 25-week-old WKY, Lean, and CP rats. (B) Relative protein expression levels of FTHFD and CA3 in liver tissues of 6- and 25-week-old WKY, Lean, and CP. All quantitative data are mean ± SEM values of four rats per group. *P < 0.05, compared with 6-week-old WKY and Lean; †*P* < 0.05, compared with 25-week-old WKY and Lean. Click here for file
