# Supplementary material for: Altered gene and protein expression in liver of the obese spontaneously hypertensive/NDmcr-cp rat
Source: Nutr Metab (Lond). 2012 Sep 21;9:87. doi: 10.1186/1743-7075-9-87 (PMC3565951; doi:10.1186/1743-7075-9-87)
Supplement: Additional file 1: Table S1 — List of significantly up-regulated genes in CP rats, compared with age-matched control rats (WKY and Lean). Table S2. List of significantly down-regulated genes in CP rats, compared with the age-matched control rats (WKY and Lean). Table S3. Comparison of gene expression in microarray analysis of proteins identified in proteomics analysis. [file 1743-7075-9-87-S1.doc]

**Supplementary Table 1.** List of significantly up-regulated genes in CP rats, compared with age-matched control rats (WKY and Lean).

| Gene name | Symbol | Log ratio | | | |
| --- | --- | --- | --- | --- | --- |
|  |  | 6-week-old | | 25-week-old | |
|  |  | WKY-CP | Lean-CP | WKY-CP | Lean-CP |
| *Lipid metabolism* |  |  |  |  |  |
| acyl-CoA synthetase long-chain family member 5 | *Acsl5* | 1.25 | 2.91 | 1.66 | 2.34 |
| cytochrome P450, family 26, subfamily A, polypeptide 1 | *Cyp26a1* | 3.45 | 4.84 | 4.01 | 2.64 |
| ELOVL family member 6, elongation of long chain fatty acids | *Elovl6* | 3.96 | 6.08 | 10.76 | 10.51 |
| fatty acid synthase | *Fasn* | 3.15 | 4.78 | 8.25 | 10.33 |
| lipocalin 2 | *Lcn2* | 2.08 | 1.18 | 2.88 | 1.52 |
| malic enzyme 1 | *Me1* | 2.60 | 3.90 | 1.92 | 3.03 |
| thyroid hormone responsive protein | *Thrsp* | 1.29 | 4.15 | 2.56 | 2.43 |
| glutathione S-transferase Yc2 subunit | *Yc2* | 4.78 | 6.72 | 5.77 | 5.78 |
| *Carbohydrate metabolism* |  |  |  |  |  |
| ATP citrate lyase | *Acly* | 1.18 | 4.00 | 2.92 | 3.94 |
| glucose-6-phosphate dehydrogenase | *G6pdx* | 1.66 | 2.23 | 1.26 | 2.20 |
| pyruvate kinase, liver and red blood cell | *Pklr* | 2.96 | 1.93 | 1.44 | 1.71 |
| solute carrier family 2, member 5 | *Slc2a5* | 3.12 | 3.38 | 3.64 | 3.25 |
| liver UDP-glucuronosyltransferase, phenobarbital-inducible form | *Udpgtr2* | 2.55 | 3.23 | 1.05 | 1.30 |
| *Protein metabolism* |  |  |  |  |  |
| histidine ammonia lyase | *Hal* | 2.37 | 1.43 | 2.04 | 1.04 |
| pyruvate dehydrogenase kinase 1 | *Pdk1* | 1.14 | 1.80 | 1.70 | 1.86 |
| *Immune system* |  |  |  |  |  |
| MIC2 like 1 | *Mic2l1* | 1.22 | 1.26 | 1.17 | 1.17 |
| pre-B-cell colony enhancing factor 1 | *Pbef1* | 1.03 | 2.44 | 1.10 | 1.77 |
| *Signal transduction* |  |  |  |  |  |
| adrenergic receptor, alpha 1 | *Adra1b* | 1.21 | 2.13 | 1.20 | 1.36 |
| homer homolog 2 | *Homer2* | 4.27 | 1.78 | 2.98 | 1.32 |
| inhibin beta E | *Inhbe* | 1.96 | 2.71 | 3.51 | 2.84 |
| retinoic acid receptor, beta | *Rarb* | 1.03 | 2.56 | 1.13 | 1.82 |
| *Others* |  |  |  |  |  |
| aquaporin 11 | *Aqp11* | 1.06 | 1.59 | 1.31 | 1.53 |
| O-6-methylguanine-DNA methyltransferase | *Mgmt* | 1.51 | 2.27 | 2.43 | 1.57 |
| nuclear protein 1 | *Nupr1* | 1.42 | 2.45 | 2.25 | 1.57 |
| reticulon 4 | *Rtn4* | 1.73 | 1.43 | 1.96 | 2.38 |

WKY; Wistar Kyoto rats, Lean; spontaneously hypertensive rats (SHR/lean), CP; SHR/NDmcr-cp (cp/cp).

**Supplementary Table 2.** List of significantly down-regulated genes in CP rats, compared with the age-matched control rats (WKY and Lean).

| Gene name | Symbol | Log ratio | | | | |  |
| --- | --- | --- | --- | --- | --- | --- | --- |
|  |  | 6-week-old | | 25-week-old | | |  |
|  |  | WKY-CP | Lean-CP | | WKY-CP | Lean-CP | |
| *Lipid metabolism* |  |  |  | |  |  | |
| annexin A6 | *Anxa6* | -1.50 | -3.07 | | -1.25 | -1.62 | |
| cytochrome P450, family 2, subfamily c, polypeptide 40 | *Cyp2c40* | -2.04 | -1.43 | | -2.16 | -1.05 | |
| cytochrome P450, family 3, subfamily a, polypeptide 11 | *Cyp3a11* | -5.61 | -6.86 | | -4.77 | -4.31 | |
| cytochrome P450, 3a18 | *Cyp3a18* | -3.87 | -4.05 | | -4.22 | -2.89 | |
| diazepam binding inhibitor-like 5 | *Dbil5* | -1.71 | -1.49 | | -2.73 | -2.72 | |
| isopentenyl-diphosphate delta isomerase | *Idi1* | -2.95 | -1.24 | | -2.38 | -3.58 | |
| sulfotransferase family, cytosolic, 1C, member 1 | *Sult1c1* | -3.88 | -4.81 | | -4.79 | -4.17 | |
| *Carbohydrate metabolism* |  |  |  | |  |  | |
| hydroxyacid oxidase 2 | *Hao2* | -4.26 | -6.02 | | -3.19 | -3.78 | |
| hydroxy-δ-5-steroid dehydrogenase, 3β- and steroid δ-isomerase 1 | *Hsd3b1* | -2.02 | -2.88 | | -2.56 | -3.37 | |
| solute carrier family 2, (facilitated glucose transporter) member 8 | *Slc2a8* | -1.21 | -1.28 | | -1.43 | -1.39 | |
| *Protein metabolism* |  |  |  | |  |  | |
| leukotriene C4 synthase | *Ltc4s* | -4.47 | -2.35 | | -6.80 | -5.13 | |
| pancreasin | *Mpn* | -2.79 | -3.94 | | -2.40 | -2.60 | |
| N-acetyltransferase 8 | *Nat8* | -3.35 | -3.39 | | -2.39 | -3.23 | |
| *Cellular amino acid metabolism* |  |  |  | |  |  | |
| flavin containing monooxygenase 1 | *Fmo1* | -1.25 | -3.46 | | -1.37 | -2.94 | |
| kynureninase (L-kynurenine hydrolase) | *Kynu* | -1.15 | -1.64 | | -2.46 | -1.07 | |
| solute carrier family 6 (neurotransmitter transporter, taurine), member 6 | *Slc6a6* | -1.53 | -3.59 | | -4.05 | -2.40 | |
| *Immune system* |  |  |  | |  |  | |
| cytokine inducible SH2-containing protein | *Cish* | -1.83 | -3.17 | | -1.61 | -1.51 | |
| growth arrest and DNA-damage-inducible 45 gamma | *Gadd45g* | -4.50 | -4.40 | | -4.48 | -4.61 | |
| glutathione S-transferase, mu 5 | *Gstm5* | -3.56 | -1.98 | | -3.23 | -1.25 | |
| SH2 domain protein 2A | *Sh2d2a* | -2.27 | -3.57 | | -1.18 | -1.30 | |
| *Cell adhesion* |  |  |  | |  |  | |
| carboxylesterase 3 | *Ces3* | -1.80 | -3.15 | | -1.45 | -2.33 | |
| insulin-like growth factor binding protein 2 | *Igfbp2* | -3.46 | -4.34 | | -6.20 | -6.05 | |
| symplekin | *Sympk* | -1.49 | -2.12 | | -1.53 | -1.67 | |
| *Signal transduction* |  |  |  | |  |  | |
| Jun oncogene | *Jun* | -2.10 | -1.68 | | -3.31 | -2.70 | |
| Ras association (RalGDS/AF-6) domain family 5 | *Rassf5* | -1.02 | -1.12 | | -2.01 | -1.42 | |
| regulator of G-protein signalling 3 | *Rgs3* | -4.39 | -3.24 | | -5.28 | -4.41 | |
| *Others* |  |  |  | |  |  | |
| cholinergic receptor, nicotinic, beta polypeptide 2 | *Chrnb2* | -2.66 | -3.29 | | -1.96 | -1.99 | |
| nasal embryonic LHRH factor | *Nelf* | -2.25 | -1.47 | | -3.17 | -2.90 | |
| nerve growth factor receptor (TNFRSF16) associated protein 1 | *Ngfrap1* | -1.84 | -1.14 | | -1.98 | -1.11 | |
| nuclear pore membrane glycoprotein 210 | *Pom210* | -2.07 | -1.48 | | -2.78 | -1.90 | |
| carrier organic anion transporter family, member 1a4 | *Slco1a4* | -2.98 | -2.86 | | -2.28 | -2.06 | |

Abbreviations as in Supplementary Table 1.

**Supplementary Table 3.** Comparison of gene expression in microarray analysis of proteins identified in proteomics analysis.

| Gene  symbol | Protein name | Log ratio | | | |
| --- | --- | --- | --- | --- | --- |
|  |  | 6-week-old | | 25-week-old | |
|  |  | WKY-CP | Lean-CP | WKY-CP | Lean-CP |
| *Lipid metabolism* | |  |  |  |  |
| CYB5A | Cytochrome b5 | 0.16 | -0.51 | -0.10 | 0.73 |
| DMGDH | Dimethylglycine dehydrogenase | 0.97 | -0.29 | -0.01 | -0.48 |
| FDPS | Farnesyl pyrophosphate synthase | -0.54 | -0.13 | -1.83 | -1.49 |
| GPD1 | Glycerol-3-phosphate dehydrogenase [NAD+] | 0.32 | 0.38 | 0.15 | 0.16 |
| MUP | Major urinary protein | - | - | - | - |
| PC | Pyruvate carboxylase | -0.66 | -0.64 | -0.52 | -0.31 |
| PDHB | Pyruvate dehydrogenase E1 component subunit beta | - | - | - | - |
| SULT1C1 | Sulfotransferase 1C1 | -3.88 | -4.81 | -4.79 | -4.17 |
| *Carbohydrate metabolism* | |  |  |  |  |
| CA3 | Carbonic anhydrase 3 | - | - | - | - |
| DLAT | Dihydrolipoyllysine-residue acetyltransferase component of pyruvate dehydrogenase complex | 1.26 | 1.88 | 2.04 | 1.93 |
| KHK | Ketohexokinase | 1.55 | 1.19 | 1.27 | 1.29 |
| MDH1 | Malate dehydrogenase | 1.44 | 1.58 | 2.88 | 1.77 |
| RGN | Regucalcin | -1.02 | -0.81 | -1.77 | -2.85 |
| *Protein metabolism* | |  |  |  |  |
| AHSG | Alpha-2-HS-glycoprotein | -1.20 | -1.69 | -1.23 | -2.07 |
| CRT | Calreticulin | -0.35 | -1.87 | -0.37 | -0.30 |
| GSTK1 | Glutathione S-transferase kappa 1 | 1.44 | 1.06 | 1.39 | 1.38 |
| HSPA5 | 78 kDa glucose-regulated protein | -0.88 | -0.70 | -0.42 | -0.56 |
| PDIA3 | Protein disulfide-isomerase A3 | - | - | - | - |
| PDIA4 | Protein disulfide-isomerase A4 | -0.03 | -0.27 | -0.13 | -0.15 |
| *Nucleic acid metabolism* | |  |  |  |  |
| FTHFD | 10-formyltetrahydrofolate dehydrogenase | 0.76 | 1.68 | 0.79 | 1.74 |
| SND1 | Staphylococcal nuclease domain-containing protein 1 | 0.36 | -0.76 | -0.55 | -0.19 |
| *Immune system process* | |  |  |  |  |
| CRP | C-reactive protein | 0.60 | 0.44 | -1.30 | -1.18 |
| HYOU1 | Hypoxia up-regulated protein 1 | 0.84 | -0.58 | -0.16 | -0.65 |
| TRA1 | Endoplasmin | -0.40 | -1.07 | -0.52 | -0.38 |
| *Others* |  |  |  |  |  |
| KRT18 | Keratin, type I cytoskeletal 18 | - | - | - | - |
| DDAH1 | N(G),N(G)-dimethylarginine dimethylaminohydrolase 1 | -0.92 | -1.29 | 0.16 | 0.40 |
| GSS | Glutathione synthetase | 0.97 | 1.43 | 1.44 | 1.81 |

Abbreviations as in Supplementary Table 1.
