# Supplementary material for: Altered gene and protein expression in liver of the obese spontaneously hypertensive/NDmcr-cp rat
Source: Nutr Metab (Lond). 2012 Sep 21;9:87. doi: 10.1186/1743-7075-9-87 (PMC3565951; doi:10.1186/1743-7075-9-87)
Supplement: Additional file 2: Figure S1 — Expression of Elovl6 and Fasn in liver tissues of 6- and 25-week-old WKY, Lean, and CP. Data are expressed relative to the mRNA expression of β-actin. All quantitative data are mean ± SEM values of six rats per group. *P < 0.05, compared with 6-week-old WKY and Lean; †P < 0.05, compared with 25-week-old WKY and Lean. Figure S2. Confirmation of the 2D-DIGE results by western blot analysis. (A) Representative immunoblot analysis of FTHFD, CA3, and β-actin (loading control) in liver tissues of representative 6- and 25-week-old WKY, Lean, and CP rats. (B) Relative protein expression levels of FTHFD and CA3 in liver tissues of 6- and 25-week-old WKY, Lean, and CP. All quantitative data are mean ± SEM values of four rats per group. *P < 0.05, compared with 6-week-old WKY and Lean; †P < 0.05, compared with 25-week-old WKY and Lean. [file 1743-7075-9-87-S2.doc]

**Supplementary Figure 1.** Expression of *Elovl6* and *Fasn* in liver tissues of 6- and 25-week-old WKY, Lean, and CP. Data are expressed relative to the mRNA expression of β-actin. All quantitative data are mean±SEM values of six rats per group. **P*<0.05, compared with 6-week-old WKY and Lean; †*P*<0.05, compared with 25-week-old WKY and Lean.

**Supplementary Figure 2.** Confirmation of the 2D-DIGE results by western blot analysis. (A) Representative immunoblot analysis of FTHFD, CA3, and β-actin (loading control) in liver tissues of representative 6- and 25-week-old WKY, Lean, and CP rats. (B) Relative protein expression levels of FTHFD and CA3 in liver tissues of 6- and 25-week-old WKY, Lean, and CP. All quantitative data are mean±SEM values of four rats per group. **P*<0.05, compared with 6-week-old WKY and Lean; †*P*<0.05, compared with 25-week-old WKY and Lean.
